# Impact of Perceived Stress and Immune Status on Decision-Making Abilities during COVID-19 Pandemic Lockdown

**DOI:** 10.3390/bs11120167

**Published:** 2021-12-02

**Authors:** Vincenza Tarantino, Ilaria Tasca, Nicoletta Giannetto, Giuseppa Renata Mangano, Patrizia Turriziani, Massimiliano Oliveri

**Affiliations:** Department of Psychology, Educational Science and Human Movement, University of Palermo, Viale delle Scienze Ed. 15, 90128 Palermo, Italy; ilariatasca@libero.it (I.T.); nicoletta.giannetto@unipa.it (N.G.); renata.mangano@unipa.it (G.R.M.); patrizia.turriziani@unipa.it (P.T.); massimiliano.oliveri@unipa.it (M.O.)

**Keywords:** stress, decision making, immune system, cognitive functions, impulsivity, COVID-19, Iowa Gambling Task, Go/No-Go, anxiety, depression

## Abstract

The ability to make risky decisions in stressful contexts has been largely investigated in experimental settings. We examined this ability during the first months of COVID-19 pandemic, when in Italy people were exposed to a prolonged stress condition, mainly caused by a rigid lockdown. Participants among the general population completed two cognitive tasks, an Iowa Gambling Task (IGT), which measures individual risk/reward decision-making tendencies, and a Go/No-Go task (GNG), to test impulsivity, together with two questionnaires, the Perceived Stress Scale and the Depression, Anxiety and Stress Scales. The Immune Status Questionnaire was additionally administered to explore the impact of the individual health status on decision making. The effect of the questionnaires scores on task performance was examined. The results showed that higher levels of perceived stress and a more self-reported vulnerable immune status were associated, separately, with less risky/more advantageous choices in the IGT in young male participants but with more risky/less advantageous choices in older male participants. These effects were not found in female participants. Impulsivity errors in the GNG were associated with more anxiety symptoms. These findings bring attention to the necessity of taking into account decision-making processes during stressful conditions, especially in the older and more physically vulnerable male population.

## 1. Introduction

Stress is a composite mental and body reaction that arises in uncontrollable situations and results in compensatory emotional and arousal responses. According to the Somatic Marker Hypothesis, emotional factors and arousal (i.e., the somatic markers) contribute and often facilitate decision-making processes [1,2]. By acting on somatic markers, stress might alter high-order cognitive functions, such as decision-making abilities [2]. This action is mediated by neurochemicals released in response to stress, such as glucocorticoids and dopamine, which all have receptors in the brain prefrontal cortex that regulate these executive functions [3,4,5,6,7,8,9,10]. Under both acute and chronic stress conditions, a shifting of executive control away from slower, more deliberative processes that largely depend on the prefrontal cortex toward more automatic, reflexive processes that mainly depend on more posterior cortical and subcortical areas, such as the amygdala, has been found [6,11,12,13]. This mechanism, which is adaptive from an evolutionary point of view, is sometimes dysregulated and might not be beneficial [14,15].

Chronic stress further complicates this scenario, since it is associated with the development of inflammatory processes, which, in turn, are responsible for brain alterations mediated by circulating inflammation markers [16,17,18]. The link between stress and inflammatory processes has become dramatically relevant after the outbreak of the COVID-19 pandemic [19]. In addition to neurological sequelae due to the viral inflammation per se, the prolonged restriction of activities, especially social ones, due to forced lockdown has generated higher levels of stress [20,21], which, in turn, might have impacted inflammatory processes and weakened the immune response to viruses [22]. According to the aforementioned literature, this prolonged stress might have affected brain functioning, especially high-order cognitive functions, such as decision making. An open question concerns the effects of immune functioning on such cognitive functions.

Importantly, sex differences have emerged in responses to stress. Physiological reactions to acute as well as chronic stress, such as alterations of the immune system and enhanced systemic inflammation (i.e., cytokines’ production), have been found to be stronger in women than in men [23]. Yet, cognitive functions under acute stress have shown sex differences, especially for what concerns decision making. Performance on the Iowa Gambling Task has revealed that higher levels of cortisol induced by acute exposure to stress were associated with a tendency toward more risk-taking choices in males, and more risk-aversive choices in females [24]. Similar sex differences in risky decision making were found when performing another version of the Iowa Gambling Task after generating anticipatory stress [10]. The pharmacologically induced increase in cortisol levels per se was found to boost risk-taking behaviors in men, whereas it had no effect in women [25]. Moreover, reward learning in men was found to be impaired after cortisol administration, while it was augmented in women [26]. While clear-cut data exist on acute stress, little is known about sex differences in decision making under chronic stress to make specific predictions [27]. Moreover, physiological reactions to both acute and chronic stress are moderated by age [23,28]. Therefore, age should be taken into account when examining cognitive responses to stress. 

The present work aimed at elucidating the impact of the individual stress levels and history of immune status on decision-making functions in the general population during a prolonged period of stress, i.e., the lockdown due to the COVID-19 pandemic. Specifically, we tested whether higher levels of stress affected the ability to make risky decisions among alternatives. To this aim, an online survey, addressed to the general population, was conducted during the first wave of the pandemic in Italy, which forced to a national-wide lockdown. Participants were asked to perform two cognitive tasks, an Iowa Gambling Task, namely, a reward-related decision-making task that measures individual tendency toward risky decisions, and a Go/No-Go task, namely, a task that measures more general behavioral impulsivity in a non-rewarded context. In addition, participants were invited to answer three questionnaires, wherein they were required to report perceived stress, depressive and anxious symptoms, as well as the presence of symptoms related to immune status functioning. We focused on the effect of chronic stress and self-reported immune status on decision making. The relationship between questionnaires scores and task performance was tested by means of multiple linear models, taking into account the moderating effects of age and sex.

## 2. Materials and Methods

### 2.1. Procedure

Both the cognitive tasks and the questionnaires were presented using an online platform, Psytoolkit [29,30] and embedded in a survey. First, socio-demographic and general health data, which include age, gender, education, and chronic diseases, were collected for each participant. Afterwards, participants were required to complete the Iowa Gambling Task and the Go/No-go task. At the end of the tasks, they were asked to fill in the Perceived Stress Scale [31,32], the Depression, Anxiety, and Stress Scales [33,34], and the Immune Status Questionnaire [35]. The screen display was adapted for different devices (computer, notebook, tablet, smartphone).

The survey was disseminated through mainstream social networks and a dedicated University website, and it was available online from 15 April to 31 May 2020. At that time, to reduce the spread of the SARS-CoV-2, Italy was adopting a rigid lockdown, lasting from 9 March to 18 May 2020, during which people were asked to move from home for essential activities only. Since 18 May 2020, these restrictions were attenuated. On 15 April, a total of 105,418 cases of COVID-19 disease in Italy were computed from the Ministry of Health, 3079 of which were hospitalized in intensive care, and 21,645 have died since the pandemic outbreak. These numbers account for the need for home confinement to reduce the spread of the virus and, on the other hand, for its psychological impact.

All data of the survey were collected, stored, and analyzed in an anonymous form. The study procedure and methods conformed to the principles embodied in the Declaration of Helsinki and were approved by the Local Ethical Committee (Comitato di Bioetica, Università di Palermo, Palermo, Italy). All respondents provided informed consent.

### 2.2. Participants

A total of 276 Italian participants fully entered sociodemographic information and performed the Iowa Gambling Task. From this sample, nine participants were excluded because they made multiple entries into the survey, one participant was excluded because of several missing responses to the task (>50%), and three participants were excluded because they made the same response repeatedly for more than 95% of trials. Among this sample, three participants did not complete the Go/No-go task. The final sample entered into separate analyses and included 260 participants who fully completed the PSS questionnaire, 251 participants who fully completed the DASS, and 250 for the ISQ. Participants’ age ranged from 19 to 69 years (M = 34.1; SD = 11.6); 67.5% were females; education was scored from 1 (elementary school) to 6 (postgraduate education) and ranged from 2 to 6 (M = 4.2; SD = 1.1); 23.7% reported a chronic disease, such as hypertension and asthma. Table S1 in Supplementary materials summarizes sociodemographic data and questionnaire scores for each subsample.

### 2.3. Cognitive Tasks

The Iowa Gambling Task (IGT) was adapted from the original version of Bechara and colleagues [1] and implemented by Psytoolkit (https://www.psytoolkit.org/experiment-library/igt.html, accessed date 13 September 2021). The original task consists of four decks of cards (A, B, C, D) among which participants have to choose one card. Each time they choose a card, they receive feedback about winning and/or losing some money. Some of the decks (A, B) are associated with a high win as well as a high loss; the other decks (C, D) are associated with a low win as well as a low loss. In the long run, decks A and B are disadvantageous/risky because they cost the most, while decks C and D are advantageous because they result in an overall gain. Participants do not know in advance the amount of money with which the card was associated, but at each choice, they receive feedback about the amount of money won and/or lost, similar to a gambling game.

In the current task, a total of 100 trials was presented. Each trial comprehended four choices, namely, four “buttons” labeled A–D. Participants were told that they had a starting bank account with EUR 2000 and that they had to try to maximize their account by choosing one of the buttons, which were associated with a chance of winning money or of having to pay a penalty. No additional information was provided, therefore, the first choices could be made casually. Buttons A and B always yielded EUR 100, whereas buttons C and D always yielded EUR 50. For each button chosen, there was a 50% chance of having to pay a penalty as well. For buttons A and B, the penalty was EUR 250, whereas for buttons C and D, the penalty was EUR 50 (see Figure S1 in Supplementary materials for an example). For every choice, the money won or lost and the updated bank amount were displayed. Trial-by-trial, participants could learn from contingencies which buttons were more advantageous or more disadvantageous/risky (learning phase). Over trials, they could decide the strategy to adopt (performance phase [10]). Each series of buttons was displayed on the screen as long as it took for the participant to make a decision. This task simulates real-life decision making in the way it weights uncertainty of reward and punishment. Poor performance on the IGT has been attributed to less sensitivity to physiological cues (somatic markers [1]), which guides risky choices in rewarded context and serves an adaptive evolutionary function.

The Go/No-go task (GNG) was adapted from a previous study [36] and implemented in Psytoolkit as the IGT. In this task, participants were presented with a series of squares on the center of the screen. The squares were either blue or red and could have one of three possible sizes (80, 100, 120 pixels), showed in a pseudo-random order. Participants were instructed to click (or touch) the blue squares (Go stimuli) as fast as possible on their appearance and to withhold the response when red squares (No-go stimuli) appeared (see Figure S2 for an example). For each trial, the deadline for response was 1000 ms. Omitted responses to Go trials or responses to No-go trials (commissions) were signaled by a feedback display (“No response” or “Error”), which was presented on the screen for 1000 ms. The task comprehended 180 trials, divided into 5 blocks. Each block contained 30 Go trials and 6 No-go trials (16.6%).

### 2.4. Questionnaires

The Perceived Stress Scale (PSS) is a self-report questionnaire that measures the degree to which some aspects of life are perceived as uncontrollable, unpredictable, and overloaded [31,32]. It refers to thoughts and feelings relative to stressful events that occurred in the last month (e.g., “In the last month, how often have you been/felt angered because of things that were outside your control?”) and includes 10 items rated on a five-point Likert scale ranging from 0 (never) to 4 (very often). The higher the total (sum) score is, the higher the degree of stress is, in other words, the participant reports less than optimal coping strategies of stress and adaptation to unpredictable situations.

The Depression, Anxiety, and Stress Scale (DASS [33,34]) is a self-report questionnaire that assesses the presence of symptoms related to depression (7 items), anxiety (7 items), and tension/stress (7 items) disorders. The questions refer to thoughts and feelings relative to the last week (e.g., “I was unable to become enthusiastic about anything”). Each item was rated on a four-point scale, from 0 (never) to 3 (very often). The higher the total (sum) score on each subscale is, the higher is the probability to have depression-, anxiety-, and/or stress-related symptoms.

The Immune Status Questionnaire (ISQ) provides a self-rated estimate of the individual immune status functioning [35]. It includes seven questions on common health complaints caused by immune system dysfunction (i.e., sudden high fever, diarrhea, headache, skin problems, muscle and joint pain, common cold, and coughing). Participants have to rate them on a five-point scale, from 0 (never) to 4 (almost always), depending on their experience in the last year. Two additional items assess the current status of general health and immune functioning, respectively, on a Likert scale from 0 (very bad) to 10 (very good). Higher total scores reflect a higher presence of symptoms related to immune deficiency disorders.

### 2.5. Data Analysis

The effect of age and sex on questionnaires raw scores were tested by separate linear regression analyses. Furthermore, questionnaires scores were correlated by a Pearson’s correlation analysis.

In the IGT, the number of times each button was selected was analyzed. Responses faster than 100 ms (anticipations) and slower than 5000 ms were excluded. The index (C + D) − (A + B) was calculated for each subject [1]. Higher scores reflect more frequent advantageous choices, whereas lower scores represent more frequent disadvantageous/risky choices. To discriminate between the learning and the performance phase, the first (50 trials) and the second part (50 trials) of the task were examined separately. Indeed, deck choices on earlier trials have been shown to be the most affected by individual differences [37] and stressors (Preston et al., 2007).

In the GNG task, the sensitivity index (*d* prime, *d′*) and response bias index (*c* criterion) were computed and analyzed, according to the Signal Detection Theory [38]. Specifically, the sensitivity index was computed on hits (correct Go responses) and false alarms (responses to No-go trials) by the formula *d′* = Z(H) − Z(FA), where Z(H) represents the z-transform of the proportion of hits (correct responses on Go trials) and Z(FA) represents the z-transform of the proportion of false alarms (wrong responses to No-go trials). The response bias was computed with the formula *c* = −(Z(H) + Z(FA))/2. A correction was applied for hits equal to the total of Go trials: (hits-0.5)/(total Go trials + 1), and for false alarms equal to 0: 0.5/(total number of No-Go trials + 1). The higher the sensitivity index is, the better is the performance. Negative response bias indicates a liberal criterion (the subject is more likely to press a button whenever a stimulus appears); positive response bias indicates a conservative criterion (the subject is less likely to press a button to a stimulus appearance). Given that feedback was delivered after errors (omission and commissions), and this could have influenced subsequent responses [39], trials anticipated by an error were removed.

Response times (RTs) were not analyzed since participants performed the survey using different devices, therefore, differences across task conditions could have influenced overall RT differences.

Statistical analyses were performed using the R software (www.r-project.org, accessed date 13 September 2021). First of all, the effects of age and sex on questionnaires scores were assessed by separate linear models (*lm* function; e.g., PSS score ~ age × sex), regardless of task performance. Then, IGT and GNG measures were fitted by means of separate multiple linear regression models, which included age and sex as predictors. In turn, a third variable was added, namely the PSS, DASS, or IQS score (e.g., Iowa score ~ age × sex × PSS score). All continuous predictors were centered (*scale* function) before entering the regression models. The models included the interaction terms in order to quantify the moderating effects of age and sex. Each DASS subscale (depression, anxiety, and stress) was entered in separate models in order to discriminate their influence on cognitive tasks. To check for the floor and ceiling effect on the dependent measure, a censored regression model was further run to confirm results (*tobit* function [40]). Models’ goodness of fit was compared based on the Akaike Information Criterion (AIC) and the Bayes information criterion (BIC); the smaller their values were, the better the fit was [41].

Additionally, with the specific intent to test the effect of stress on learning from contingencies and on learning over time, we analyzed performance on a trial-by-trial bases. To this end, the task was divided into 4 blocks of 25 trials each [10] and participants were categorized as perceiving low versus high chronic stress based on a median split of their PSS score [27]. A generalized linear Mixed-Effects model was performed by means of the *glmer* function of the *lme4* R package [42]. In the model, the proportion of advantageous decks (C or D vs. A or B) was entered as dependent variable, while predictors were the number of block, the presence of a fee to pay in the trial (penalty), and the low or high PSS score. The presence of a fee in the previous trial was entered as covariate, as follows: *glmer* (proportion of advantageous choices ~ block × penalty × PSS + preceding penalty + (block | subj)). The random structure included random intercepts and slopes for block number (block), correlated by participant (subj).

## 3. Results

### 3.1. Effects of Age and Sex on Questionnaires Scores

Table 1 shows the means and the standard deviations of questionnaires scores, divided by age and sex. Although in the regression analysis age was entered as continuous variable, for the sake of clarity data in this descriptive table were split by the mean age (i.e., 34 years).

The effect of age and sex on questionnaires scores were tested by separate linear regression analyses, one for each questionnaire. A significant effect of age on the stress subscale of DASS (*b* = −1.70, *SE* = 0.79, *t* = −2.14, *p* = 0.033) and on the ISQ score (*b* = −1.01, *SE* = 0.31, *t* = −3.24, *p* < 0.001) was found. Surprisingly, younger participants reported more stress and immune-related symptoms than older participants. A significant sex effect was found in the PSS (*b* = −3.33, *SE* = 0.9, *t* = −3.67, *p* < 0.001), in the anxiety (*b* = −2.3, *SE* = 0.97, *t* = −2.37, *p* = 0.018) and stress (*b* = −3.94, *SE* = 1.22, *t* = −3.22, *p* = 0.001) subscales of DASS, and in the ISQ (*b* = −1.6, *SE* = 0.48, *t* = −3.31, *p* = 0.001). Namely, female participants perceived higher levels of stress than males and reported more anxiety, stress, and immune-related symptoms. No significant age × sex interactions emerged. No effects on the one-items on current general health and current immune functioning were found (*p* > 0.086).

All questionnaires scores significantly and positively correlated to each other (see Table S2 in Supplementary materials).

### 3.2. Iowa Gambling Task (IGT)

Table 2 summarizes the mean number of times the participants chose advantageous buttons (C, D) and disadvantageous ones (A, B). The total mean IGT score was 7.29 (SD = 36.86).

**Figure 1:**
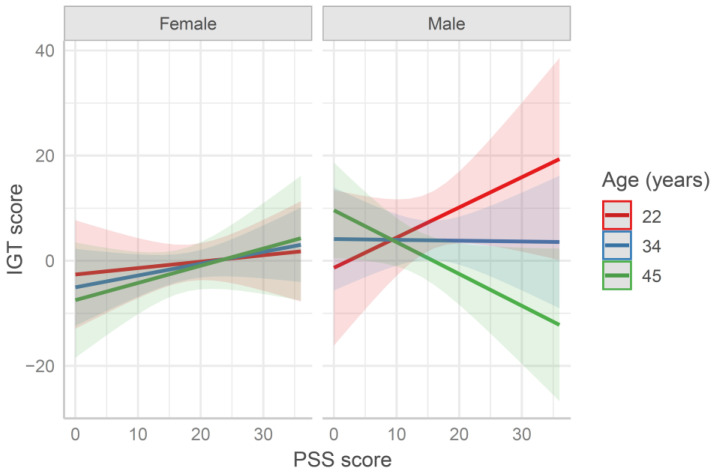
Predicted Iowa Gambling (IGT) score in the first 50 trials fitted by the model age × sex × Perceived Stress Scale (PSS) score. Values at mean age ± 1 SD are reported for representation purposes (in the model age was entered as continuous variable). The higher the PSS score is, the higher the perceived stress is. The higher the IGT score is, the higher the advantageous choices are.

The IGT scores were fitted by the age × sex × PSS multiple regression model (see Table S3 in Supplementary materials). This analysis yielded a significant main effect of sex on the scores on the first 50 trials of the IGT and a three-way interaction (*b* = −4.79, *SE* = 2.26, *t* = −2.12, *p* = 0.035). Overall, in line with past findings [43,44], female participants performed slightly worse than male ones in the IGT. In order to qualify this interaction, participants were split into female and male groups. While the IGT score of the male group was significantly affected by age (*b* = −4.74, *SE* = 2.1, *t* = −2.26, *p* = 0.026) and age × PSS (*b* = −4.08, *SE* = 1.97, *t* = −2.07, *p* = 0.042), the IGT score of the female group was not affected by age and/or PSS score (all *ps* > 0.2). Figure 1 shows the model’s plot, namely, the effect of the interaction term is represented (*sjPlot* R package [45]). As evident, in the male group, younger participants with higher PSS scores showed higher IGT scores, whereas older participants with higher PSS scores showed lower IGT scores.

**Figure 2:**
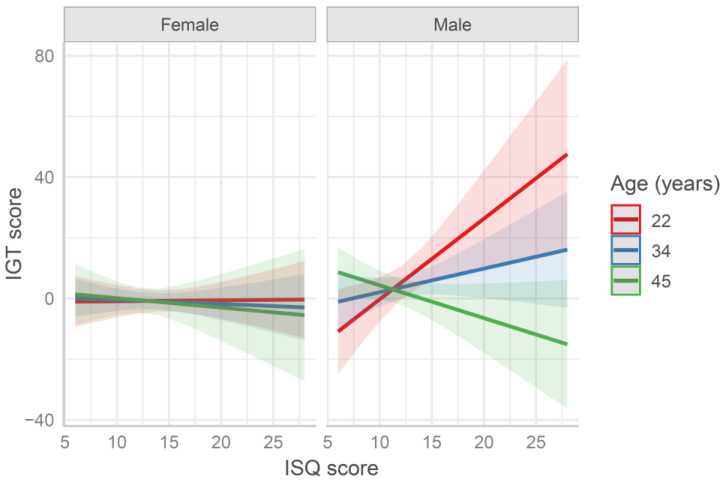
Predicted Iowa Gambling (IGT) score in the first 50 trials fitted by the model age × sex × Immune Status Questionnaire (ISQ) score. Values at mean age ± 1 SD are reported for representation purposes (in the model age was entered as continuous variable). The higher the ISQ score is, the worse the individual immune status is. The higher the IGT score is, the higher the advantageous choices are.

When the IGT scores were fitted by a model including the ISQ score (IGT ~ age × sex × ISQ, see Table S3), a significant effect of sex emerged (*b* = 5.66, *SE* = 2.33, *t* = 2.43, *p* = 0.016), in addition to a three-way interaction as well (*b* = −6.43, *SE* = 2.8, *t* = −2.3, *p* = 0.022). When analyzing females and males separately, a significant effect of age (*b* = −4.56, *SE* = 2.27, *t* = −2.01, *p* = 0.047) and an age × ISQ interaction (*b* = −7.07, *SE* = 2.47, *t* = −2.87, *p* = 0.005) was found, in the male group only. As represented in Figure 2, younger participants who reported higher ISQ scores (i.e., more frequent symptoms related to the immune system) obtained higher IGT scores (i.e., more advantageous choices), whereas older participants who reported higher ISQ scores were those who obtained lower IGT scores.

To explore the interaction of perceived stress and reported immune status on the Iowa task, both the PSS and ISQ scores were entered into the model. To reduce the number of all possible interaction factors with age and sex, a backward stepwise selection of predictors was performed (*stepAIC* function of the MAAS R package [46]). The results are shown in Table S3. In addition to the main effect of sex (*b* = 5.84, *SE* = 2.36, *t* = 2.47, *p* = 0.016) and age × sex (*b* = −5.2, *SE* = 2.58, *t* = −2.02, *p* = 0.045), the age × sex × ISQ was significant (*b* = −5.65, *SE* = 2.86, *t* = −1.97, *p* = 0.049). On the other hand, the interaction between PSS and ISQ did not yield a significant effect. Since the two questionnaires scores correlated, we might infer that their impact on the IGT score had the same direction. However, these results should be taken with caution because the sample size may have been limited to detect the interaction between perceived stress and reported immune status moderated by age and sex [47].

As shown in Table S4 of Supplementary materials, the model age × sex × ISQ is the one that explains the IGT score with the best goodness of fit.

The IGT performance in the first 50 trials was not significantly influenced by scores on DASS depression, anxiety, and stress subscales. Remarkably, none of the predictors significantly affected the IGT score when the last 50 trials were entered in the model as a dependent variable. Furthermore, the reduced model age × sex did not significantly fit the IGT data.

The learning effect was confirmed in all participants, namely, the trial-by-trial analysis showed that the proportion of advantageous choices significantly increased from block 1 to block 4 (all *ps* < 0.004). Participants who perceived low stress showed a steeper learning effect (in block 2 vs. 1 *p* = 0.018, and in block 3 vs. 1, *p* = 0.042). Overall, the presence of a fee to pay in the current or in the preceding trial did not affect choices (*p* = 0.381 and *p* = 0.239, respectively). However, compared to block 1 (reference level), the low-stressed group learned to choose advantageous buttons more from penalty than from the absence of fee, whereas the high-stressed group learned from penalty in block 1, then from the absence of fee or was not affected by it. Table S7 and Figure S4 reported full results.

Overall, the IGT performance when executing the task on a computer or notebook (*n* = 110) did not statistically differ from performance when executing the task on a tablet or smartphone (*n* = 150) (first 50 trials: *t* = 1.59, *p* = 0.112; last 50 trials: *t* = 1.93, *p* = 0.055).

### 3.3. Go/No-Go Task (GNG)

The mean *d′* value (sensitivity index) was 3.69 (SD = 0.6); the mean *c* criterion was −0.37 (SD = 0.28); the mean of response time on Go trials was 462 ms (SD = 82).

**Figure 3:**
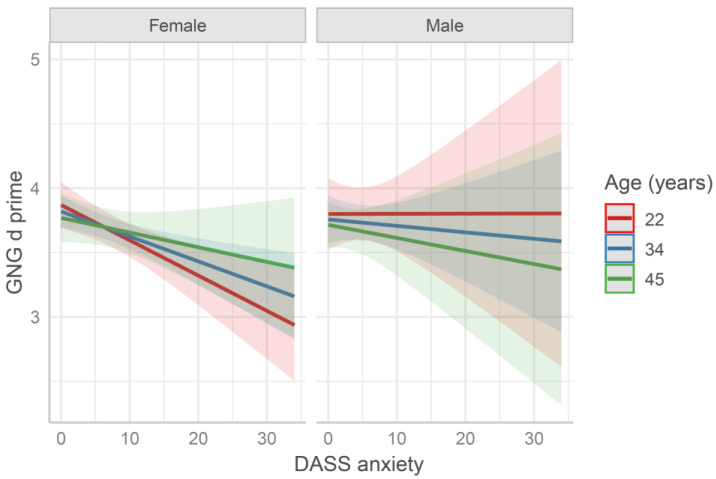
Predicted Go/No-Go (GNG) performance fitted by the model age × sex × DASS anxiety score. Values at mean age ± 1 SD are reported for representation purposes (in the model age was entered as continuous variable). The higher the *d* prime values (*d′*) are, the better the performance is.

The *d′* data were fitted by separate models containing both age and sex as predictors, plus a questionnaire score (see Table S5 and Figure 3). The model age × sex × DASS anxiety subscale yielded a significant effect of the anxiety score (*b* = −0.14, *SE* = 0.04, *t* = −3.12, *p* = 0.002). Namely, participants with higher anxiety scores presented lower *d′* values. The other DASS subscales and the other questionnaires did not significantly predict the *d′* data. Moreover, the reduced model age × sex did not yield a significant fitting.

The model that significantly predicted the *c* criterion values was age × sex (see Table S6). Specifically, an age effect emerged (*b* = 0.07, *SE* = 0.02, *t* = 2.96, *p* = 0.003) that revealed that older participants adopted a higher criterion, in other words, were more prudent in responding (i.e., lower false alarms). No significant interaction was found.

The type of device used for executing the task did not affect *d′* values (*t* = −0.735, *p* = 0.463), whereas the *c* criterion was lower (more liberal) when using a computer or a notebook compared to a tablet or a smartphone.

## 4. Discussion

The present study was aimed at investigating the impact of the individual stress levels and immune status on decision-making functions, in a time period characterized by prolonged stress exposure in the general population, i.e., during a rigid lockdown imposed in Italy at the first wave of the COVID-19 pandemic. The results revealed that the perceived stress in the last month and the self-assessed immune status affected, separately, decision-making abilities, in a different manner according to age and sex. Namely, although the female participants reported overall higher levels of perceived stress and more stress-related symptoms, in line with recent evidence [48], decision-making abilities were influenced by perceived stress levels and immune status in the male participants only. Specifically, older men who reported higher levels of stress or more vulnerable immune status made less advantageous/more risky choices (i.e., they lost more money), whereas younger males who reported higher levels of stress or more vulnerable immune status made more advantageous/less risky choices (i.e., they gained more winnings).

These results extend past evidence showing that decision making in female and male participants is differentially affected by acute stress, with female participants performing better and male participants performing worse [10,23,24]. In particular, they confirm previous studies that found that stress amplifies sex differences in the use of strategies during risky decisions, since males made more risk-taking choices and females made more risk-aversive choices [24,49]. Furthermore, they retrace studies that have shown that behavioral performance in a monetary reward task was impaired under induced high cortisol concentrations in men, while it was improved in women [26].

From a neural point of view, sex and age differences could be explained in the light of different neurobiological stress-induced effects on prefrontal cortex (PFC) functioning. Indeed, chronic stress was found to produce structural changes in PFC architecture of pyramidal cells [50]. Under optimal, stress-free conditions, the PFC works to inhibit inappropriate responses and allows nuanced decision making [50,51]. Instead, chronic stress impacts several PFC functions, such as extracting contingencies, elaborating abstract rules, and developing strategies addressed to the accomplishment of a decision [52]. Moreover, acute as well as chronic stress causes an alteration of baseline dopamine levels, in young as well as in older adults [53,54,55]. Dopamine levels in the PFC are crucial for the maintenance of stimulus–outcomes associations, a function required in the learning phase of the IGT [56]. Laboratory studies have shown that female estrogens raise baseline dopamine signaling in response to stress [53]. Therefore, we might infer that PFC functioning in females was more preserved under stress conditions. The exposure to stress might have interacted with aging-related PFC reduction of dopaminergic reserve and could have altered reward sensitivity [57], leading to suboptimal choices in older male adults.

The present work extends previous studies that investigated the effects of acute stress on reward-related decision making e.g., [8] and the effects of receiving feedback during the task (winnings or losses) on driving subsequent choice behavior [58,59], by demonstrating that during a prolonged environmental stress exposure in a real context, the most detrimental effect of stress was on older and more stressed male participants. More importantly, it adds evidence that the immune status significantly interacted with age and sex in modulating decision making. In line with previous findings showing that body inflammation states are associated with behavioral preferences for immediate versus delayed rewards [60], older male participants who self-reported more immune deficits showed a tendency toward more risky, but disadvantageous in the long run, choices. The statistical analyses showed that the model including the immune status data better fit the decision-making performance than the model including perceived stress. This result opens new perspectives in taking into account the effect of immune functioning on higher-order cognitive functions.

On the other hand, young male participants, who overall reported higher levels of stress than older male adults, were protected against disadvantageous/risky decision making. In particular, younger males who reported symptoms of impaired immune systems were also those who made more conservative/cautious choices in the task. This suggests that the higher perception of vulnerability to health issues (i.e., reporting more immune status problems) could counteract the tendency towards risky decisions under stress in younger ages. Of note, the subjective perception of uncontrollability, unpredictability, and overloading events (i.e., the PSS scores) rather than the presence of stress-related symptoms per se (i.e., the DASS scores) affected decision behavior. This finding highlights the importance of considering subjective perceptions more than the objective stress exposure.

All the observed effects on the IGT performance concerned the first part (50 trials) of the task, that is, the learning phase, when participants have to learn stimuli–reward contingencies, to catch regularities, and to adjust their choice in order to maximize gains. No significant effects emerged when the score on the last part of the task was analyzed. Furthermore, we observed a steeper learning effect in low-stressed participants and a different pattern of learning from penalty over time compared to high-stressed participants. These results are in line with previous investigations on acute stress exposure, documenting that cortisol alters sensitivity to both positive and negative feedback during the learning phases of a probabilistic reward task, i.e., it generates more inflexible adjustments of choice behavior to reward [26,49,61]. Moreover, previous studies showed that subjects with higher acute stress levels are slower in learning the contingencies of the IGT and need longer times to shift toward advantageous decision making [10]. Accordingly, we might hypothesize that the poorer performance of older participants, with higher levels of chronic stress or more vulnerable immune status, could be due to an inadequate learning from contingencies process.

With regard to the inhibitory ability, the GNG performance was affected by the score on the anxiety subscale of the DASS, irrespective of age and sex. In other words, the higher the participant reported anxiety symptoms, the higher the commission (impulsive) errors were. A similar positive correlation between all the DASS subscales and impulsivity, as measured by a self-reported questionnaire (Barratt Impulsiveness Scale, BIS-11), was found across a wide age range [62]. In addition, laboratory-induced anxiety has been shown to diminish reactive inhibitory control [63].

Unlike the IGT performance, the GNG performance was not affected by stress and self-reported immune status. This dissociation suggests that these factors specifically influenced complex executive functions more than motor inhibition. Furthermore, it could reflect the involvement of distinct neural circuits within the PFC: the ventromedial and orbitofrontal brain regions that mediate reward-related decision-making tasks [64,65], which are also more involved in modulating immune responses, such as in cytokines neuromodulation [66], compared to inferior and dorsal prefrontal regions that mediate motor inhibition [67]. There is also evidence for a differential role of each hemisphere in mediating decision making and Go/No-go functions as well as stress and immune responses [68,69]. Future studies are needed to clarify the neural dynamics of response inhibition and decision making under prolonged stress or immune deficiency circumstances [70,71].

Overall, the study results must be interpreted in light of some limitations. First of all, no survey questions were addressed to test stress and health issues specifically related to COVID-19 infection. Therefore, we could not quantify the direct or indirect impact of COVID-19 infection per se, which could explain the immune status and possibly the stress level. This is especially true for older people who could have been more affected by job loss, household situation, and loss of loved persons. Secondly, given that the type of stress investigated here is more equivalent to a chronic than an acute state, the results could only partially be comparable to experimental research that has examined the effects of stress in laboratory settings.

## 5. Conclusions

Based on the present findings, we might conclude that higher levels of prolonged stress and altered immune functioning both impacted, separately, decision-making abilities in men but not in women, by protecting them against risky choices in younger ages and by making them more vulnerable to risky choices in older ages. The study enriches previous research on the effects of acute stress exposure in laboratory settings on decision making, encourages future investigations on the effects of environmental stress, and emphasizes the need to further clarify the role of age and sex. Importantly, it contributes to the research that aims at investigating the association between cognitive skills and the immune system functioning. Furthermore, since the study was conducted during the pandemic lockdown, our results highlight the importance of assessing choice behaviors during such critical condition.

## Figures and Tables

**Table 1:** Mean (and standard deviation) scores on questionnaires. Perceived Stress Scale, PSS; Depression, Anxiety, and Stress Scales; DASS; Immune Status Questionnaire, ISQ.

	Age < 34 y		Age > 34 y	
	F	M	F	M
PSS	18.8 (6.5)	16.1 (6)	18.2 (6.9)	13.3 (7)
DASS Depression	12.3 (8.8)	11.3 (9)	10.1 (10.5)	7.9 (8.8)
DASS Anxiety	7.7 (7.8)	5.1 (4.7)	6 (7.2)	3.7 (6.5)
DASS Stress	17.1 (9.6)	12.4 (7.2)	14.6 (8.7)	11.3 (8.8)
ISQ	14.9 (3.8)	12.8 (2.8)	12.6 (3)	11.7 (3)
Current General Health	7.8 (1.1)	8 (1.2)	7.5 (1.1)	7.8 (1)
Current Immune Functioning	7.7 (1.4)	8.1 (1.3)	8.1 (1.2)	8.3 (1.2)

**Table 2:** Mean (SD) number of choices, expressed in percentage, and response times (RTs), expressed in milliseconds, in the Iowa Gambling Task.

	Button A or B		Button C or D	
	% Choice	RTs	% Choice	RTs
First 50 Trials	49.3 (17.3)	1319 ms (581)	50.7 (17.3)	1225 ms (560)
Last 50 Trials	43.9 (23.6)	930 ms (487)	56.1 (23.6)	847 ms (439)

## Data Availability

The data presented in this study are available on request from the corresponding author.

## Supplementary Materials

The following are available online at https://www.mdpi.com/article/10.3390/bs11120167/s1, Figures S1–S4; Tables S1–S7.
