# Supplementary material for: Impact of Perceived Stress and Immune Status on Decision-Making Abilities during COVID-19 Pandemic Lockdown
Source: Behav Sci (Basel). 2021 Dec 2;11(12):167. doi: 10.3390/bs11120167 (PMC8698277; doi:10.3390/bs11120167)
Supplement: Supplementary file 1 [file behavsci-11-00167-s001.zip › behavsci-1398865-supplementary.pdf]

Supplementary materials

Figure S1. An example of two trials of the Iowa Gambling Task (IGT). The starting bank amount was always displayed in the upper corner of the screen and trial-by-trial updated. Buttons A and B were associated with winnings of EUR 100 or penalties of EUR 250, whereas buttons C and D were associated with winnings of EUR 50 or penalties of EUR 50.

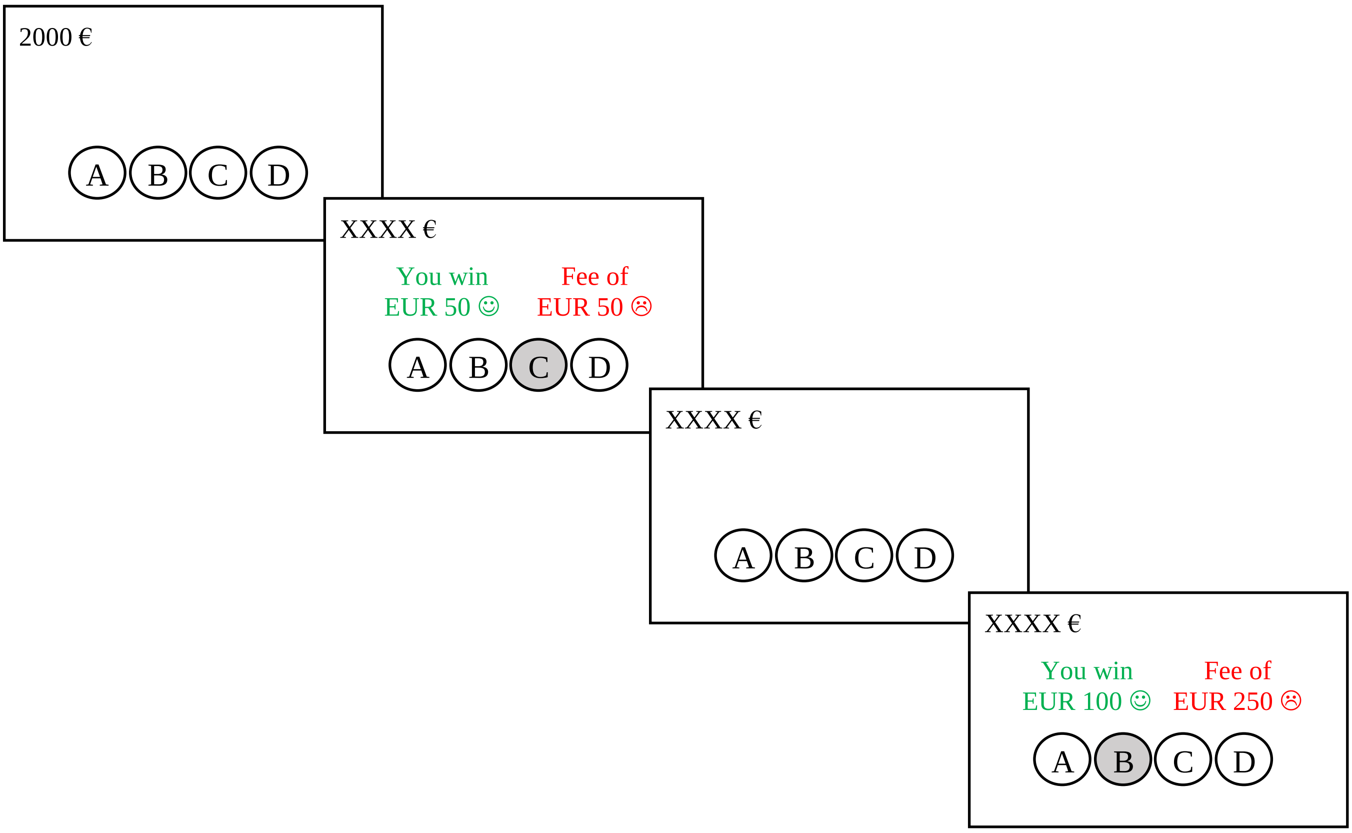

Figure S2. An example of four trials of the Go/No-go task (GNG). The blue square was the Go signal, whereas the red square was the No-go signal. The square size (80, 100 or 120 pixel) was counterbalanced across the Go and No-Go conditions. Each trial lasted 1000 ms.

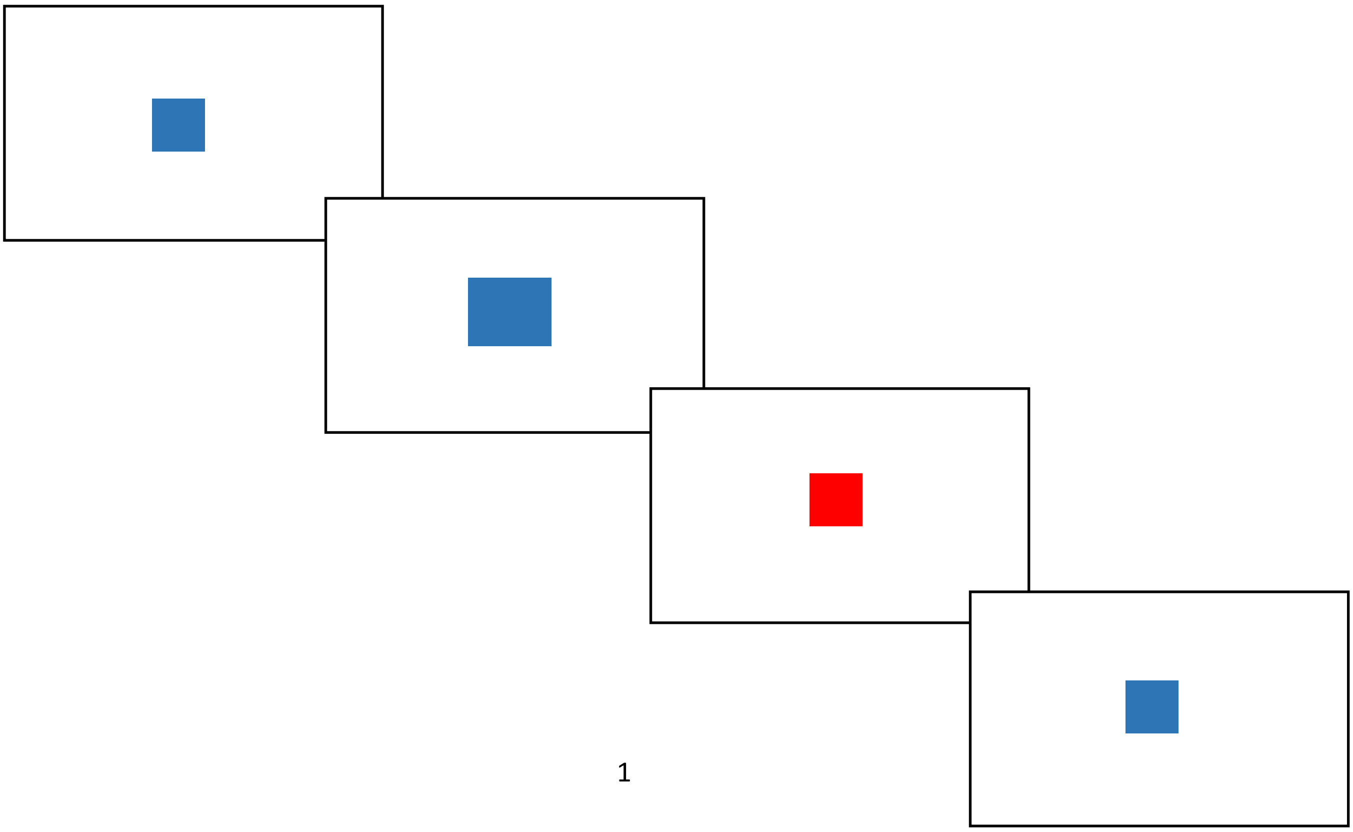

Figure S3. Distribution of age in the study sample, grouped by sex. The blue line represents mean age; the dashed red line represents 1 SD below mean; the dashed green line represents 1 SD above the mean.

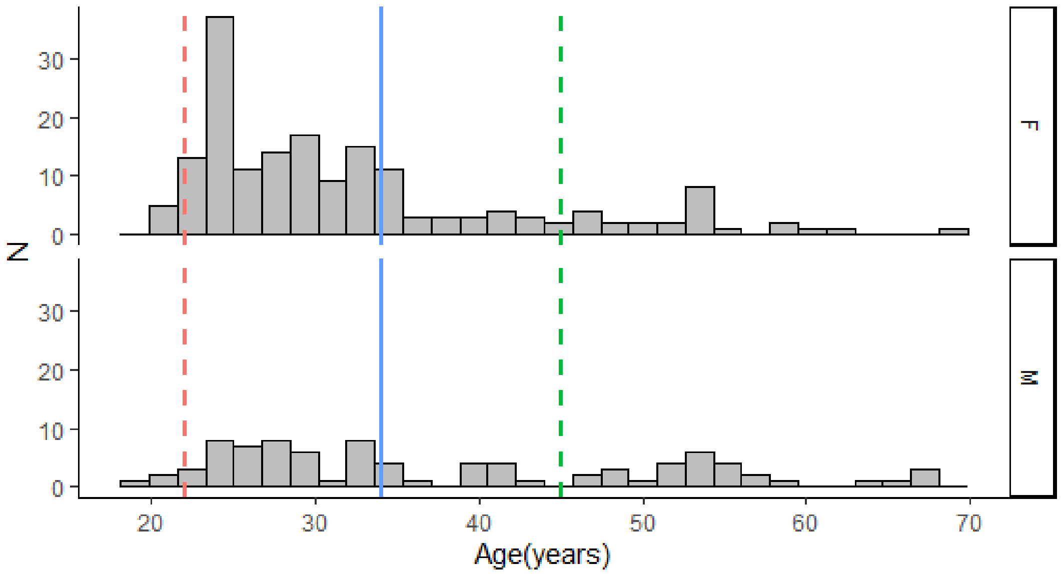

Figure S4. Proportion of advantageous choices (C or D vs. A or B buttons) as predicted by the model in Table S7.

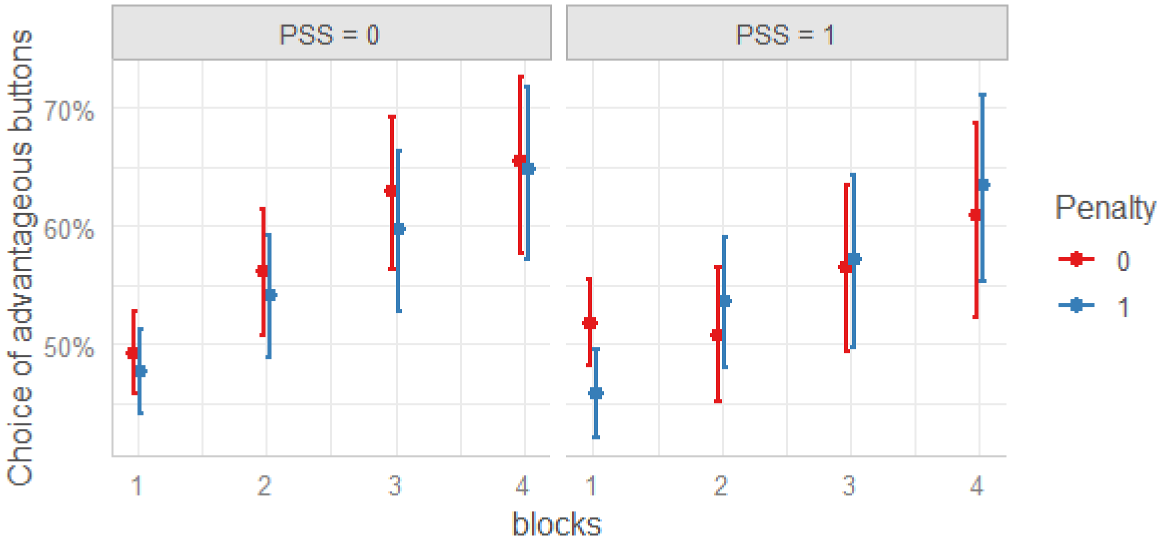

Table S1. Demographics of participants who completed the Perceived Stress Scale (PSS), the Depression, Anxiety and Stress Scales (DASS), and the Immune Status Questionnaire (ISQ).

	**PSS**	**DASS**	**ISQ**
N	260	251	250
Mean age (SD), years	34.3 (11.6)	33.9 (11.2)	33.9 (11.2)
Female	66.9 %	66.9 %	66.8 %
Education (SD), level	4.2 (1.1)	4.3 (1.1)	4.3 (1.1)
Chronic disease	23.7 %	24.6 %	24.7 %

Table S2. Pearson's correlation coefficients between questionnaires scores. PSS = Perceived Stress Scale, DASS = Depression, Anxiety and Stress Scales, ISQ = Immune Status Questionnaire.****p* < 0.001

	**PSS**	**DASS depression**	**DASS anxiety**	**DASS stress**	**ISQ**
**PSS**	1				
**DASS depression**	.702***	1			
**DASS anxiety**	.573***	.643***	1		
**DASS stress**	.757***	.72 ***	.622***	1	
**ISQ**	.323***	.317***	.369***	.346***	1

Table S3. Results of the multiple linear regression models that significantly fitted the Iowa score in the first 50 trials (IGT). Estimates = beta coefficients; CI = confidence interval; Sex: 1 = female, 2 = male; PSS = Perceived Stress Scale; ISQ = Immune Status Questionnaire.

**Predictors**	**Estimates**	***CI***	***t***	***p***
Model: IGT ~ Age × Sex × PSS				
(Intercept)	-1.16	-3.73 – 1.42	-0.89	0.376
Age	-0.66	-3.53 – 2.21	-0.45	0.651
Sex	5.07	0.42 – 9.73	2.15	**0.033**
PSS	1.55	-1.05 – 4.15	1.17	0.242
Age × Sex	-4.08	-8.72 – 0.56	-1.73	0.084
Age × PSS	0.71	-2.14 – 3.56	0.49	0.625
Sex × PSS	-1.60	-6.47 – 3.26	-0.65	0.516
Age × Sex × PSS	-4.79	-9.24 – -0.34	-2.12	**0.035**
Model: IGT ~ Age × Sex × ISQ				
(Intercept)	-0.90	-3.51 – 1.71	-0.68	0.496
Age	-0.10	-3.06 – 2.86	-0.07	0.947
Sex	5.66	1.06 – 10.26	2.43	**0.016**
PSS	-0.54	-3.30 – 2.23	-0.38	0.703
Age × Sex	-4.46	-9.37 – 0.45	-1.79	0.075
Age × ISQ	-0.65	-4.14 – 2.84	-0.37	0.715
Sex × ISQ	3.25	-2.02 – 8.52	1.22	0.225
Age × Sex × ISQ	-6.43	-11.93 – -0.92	-2.3	**0.022**
Model: IGT ~ Age × Sex × PSS × ISQ				
(Intercept)	-1.39	-4.05 – 1.28	-1.02	0.308
Age	-0.25	-3.20 – 2.71	-0.16	0.870

Sex	5.84	1.20 – 10.48	2.47	**0.014**
PSS	1.89	-0.42 – 4.21	1.6	0.111
ISQ	-1.11	-3.97 – 1.75	-0.76	0.447
Age × Sex	-5.2	-10.26 – -0.15	-2.02	**0.045**
Age × PSS	0.34	-2.14 – 2.83	0.27	0.786
Age × ISQ	-1.07	-4.60 – 2.46	-0.59	0.552
Sex × ISQ	3.27	-2.11 – 8.65	1.19	0.235
PSS × ISQ	1.25	-1.00 – 3.51	1.09	0.277
Age × Sex × ISQ	-5.65	-11.27 – -0.04	-1.97	**0.049**
Age × PSS × ISQ	2.21	-0.29 – 4.71	1.74	0.084

Table S4. Comparison among models fitting the score in the first 50 trials (function *compareLM* of the *rcompanion* R package, Mangiafico, 2015). AIC = Akaike information criterion; BIC = Bayes information criterion; Adj R^2 = Adjusted R^2.

**Model**	**AIC**	**AICc**	**BIC**	**R^2**	**Adj R^2**	***F***	**df**	***p***
Age × Sex × PSS	2210	2211	2242	0.055	0.029	2.11	7,252	0.042
Age × Sex × ISQ	2106	2107	2138	0.066	0.04	2.47	7,242	0.018
Age × Sex × PSS × ISQ	2109	2110	2155	0.087	0.045	2.07	11,238	0.023

Table S5. Results of the multiple linear regression model that significantly fitted the sensitivity index (*d'*) of the Go/No-go task. Estimates = beta coefficients; CI = confidence interval; Sex: 1 = female, 2 = male; DASS = Depression, Anxiety and Stress Scales.

**Predictors**	**Estimates**	***CI***	***t***	***p***
Model: *d'*~ Age × Sex × DASS anxiety				
(Intercept)	3.70	3.60 – 3.79	78.64	**<0.001**
Age	0.00	-0.10 – 0.10	0.02	0.985
Sex	0.03	-0.14 – 0.19	0.32	0.746
DASS anxiety	-0.14	-0.22 – -0.05	-3.12	**0.002**
Age × Sex	-0.08	-0.26 – 0.11	-0.82	0.415
Age × DASS anxiety	0.06	-0.04 – 0.15	1.22	0.223
Sex × DASS anxiety	0.10	-0.09 – 0.29	1.03	0.303
Age × Sex × DASS anxiety	-0.10	-0.32 – 0.13	-0.84	0.404
R^2/ Adjusted R^2	0.058/0.031			
F	2.15			
df	7, 244			
p	0.039			

Table S6. Results of the multiple linear regression model that significantly fitted the *c* criterion values of the Go/No-go task. Estimates = beta coefficients; CI = confidence interval; Sex: 1 = female, 2 = male.

**Predictors**	**Estimates**	***CI***	***t***	***p***
Model: *c* ~ Age × Sex				
(Intercept)	-0.38	-0.42 – -0.34	-17.38	**<0.001**
Age	0.07	0.02 – 0.12	2.96	**0.003**
Sex	0.02	-0.06 – 0.09	0.48	0.632

Age × Sex	-0.03	-0.10 – 0.04	-0.88	0.379
R^2/ Adjusted R^2	0.046/0.034			
F	4.07			
df	3,256			
p	0.007			

Table S7. Results of the multiple linear regression model that fitted the proportion of choices of button C and D (level 1) relative to A and B (level 0). Trial-by-trial responses were entered. Dichotomous predictors were included in the model, expect block. The interaction between the number of the block (from 1 to 4), the presence (level 1) or absence (level 0) of a fee to pay (penalty), and a high (> 17, level 1) or low (≤ 17, level 0) PSS score was assessed. The presence (level 1) or absence (level 0) of a penalty in the preceding trial was entered as covariate. Random intercepts and slopes for block number (block), correlated by participant (subj), were considered. PSS = Perceived Stress Scale; CI = confidence interval.

**Model**	***Odds Ratios***	***CI***	***z***	***p***
Proportion of advantageous choices ~ block × penalty × PSS + preceding penalty + (block | subj)				
(Intercept)	0.97	0.84 – 1.11	-0.432	0.666
Block 2	1.32	1.10 – 1.58	2.994	**0.003**
Block 3	1.75	1.37 – 2.24	4.445	**<0.001**
Block 4	1.96	1.44 – 2.66	4.270	**<0.001**
Penalty	0.94	0.81 – 1.08	-0.876	0.381
PSS	1.11	0.91 – 1.35	1.021	0.307
Preceding penalty	1.03	0.98 – 1.09	1.178	0.239
Block 2 × penalty	0.98	0.80 – 1.20	-0.187	0.852
Block 3 × penalty	0.93	0.75 – 1.15	-0.652	0.514
Block 4 × penalty	1.03	0.83 – 1.28	0.297	0.767
Block 2 × PSS	0.73	0.56 – 0.95	-2.371	**0.018**
Block 3 × PSS	0.69	0.48 – 0.99	-2.037	**0.042**
Block 4 × PSS	0.74	0.47 – 1.15	-1.344	0.179
Penalty × PSS	0.84	0.68 – 1.03	-1.707	0.088
Block 2 × penalty × PSS	1.45	1.08 – 1.95	2.458	**0.014**
Block 3 × penalty × PSS	1.40	1.04 – 1.91	2.185	**0.029**
Block 4 × penalty × PSS	1.38	1.02 – 1.89	2.059	**0.039**
Marginal R^2 / Conditional R^2	0.012 / 0.357			

Mangiafico, S.S. 2015. An R Companion for the Handbook of Biological Statistics. https://rcompanion.org/rcompanion/
